# Drug Repurposing for AML: Structure-Based Virtual Screening and Molecular Simulations of FDA-Approved Compounds with Polypharmacological Potential

**DOI:** 10.3390/biomedicines13112605

**Published:** 2025-10-24

**Authors:** Mena Abdelsayed, Yassir Boulaamane

**Affiliations:** 1 Lankenau Institute for Medical Research, Philadelphia, PA 19096, USA; 2 Laboratory of Innovative Technologies, National School of Applied Sciences of Tangier, Abdelmalek Essaadi University, Tetouan 93000, Morocco; boulaamane.yassir@etu.uae.ac.ma

**Keywords:** acute myeloid leukemia (AML), drug repurposing, polypharmacology, structure-based virtual screening, molecular dynamics simulation, LSD1 (KDM1A), BCL-2, IDH1 R132H, multitarget therapeutics, ADMET profiling

## Abstract

**Background:** Acute myeloid leukemia (AML) is a heterogeneous hematologic malignancy characterized by impaired differentiation, apoptosis resistance, and metabolic reprogramming, which collectively contribute to therapeutic resistance and poor clinical outcomes. While targeted agents—such as LSD1 inhibitors, the BCL-2 inhibitor venetoclax, and IDH1 inhibitors—have provided clinical benefit, their efficacy is often limited by compensatory signaling and clonal evolution. This study aimed to identify FDA-approved compounds with multitarget potential to simultaneously modulate key epigenetic, apoptotic, and metabolic pathways in AML. **Methods:** Structure-based virtual screening of 3957 FDA-approved molecules was performed against three AML-relevant targets: lysine-specific demethylase 1 (LSD1), BCL-2, and mutant IDH1 (R132H). Top-ranked hits were evaluated using ADMET prediction and molecular dynamics (MD) simulations to assess pharmacokinetic properties, toxicity, and ligand–protein complex stability over 100 ns trajectories. **Results:** Three compounds—DB16703, DB08512, and DB16047—exhibited high binding affinities across all three targets with favorable pharmacokinetic and safety profiles. MD simulations confirmed the structural stability of the ligand–protein complexes, revealing persistent hydrogen bonding and minimal conformational deviation. These findings suggest that these repurposed drugs possess a promising multitarget profile capable of addressing AML’s multifactorial pathophysiology. **Conclusions:** This computational study supports the feasibility of a polypharmacology-based strategy for AML therapy by integrating epigenetic modulation, apoptotic reactivation, and metabolic correction within single molecular scaffolds. However, the identified compounds (Belumosudil, DB08512, and Elraglusib) have not yet demonstrated efficacy in AML models; further preclinical validation is warranted to substantiate these predictions and advance translational development.

## 1. Introduction

Acute Myeloid Leukemia (AML) remains one of the most aggressive hematologic malignancies, often marked by poor prognosis and therapeutic resistance, especially in patients ineligible for high-dose chemotherapy [1]. The emergence of targeted therapies, such as inhibitors of IDH1/2 and BCL-2, has begun reshaping the treatment landscape by promoting differentiation and apoptosis of leukemic blasts [2]. For example, in acute promyelocytic leukemia (a distinct AML subtype), treatment with all-trans retinoic acid (ATRA) plus arsenic trioxide (ATO) achieves high cure rates by targeting the PML-RARα fusion oncoprotein for degradation. This landmark success illustrates the potential of precision therapies in AML, though ATO resistance can emerge via mutations in PML [3,4].

Lysine-specific histone demethylase-1A (LSD1, also known as KDM1A) is a critical epigenetic regulator that maintains differentiation blocks in AML. Inhibition of LSD1 induces differentiation of leukemic cells, particularly when combined with agents like all-trans retinoic acid (ATRA), and is currently under clinical investigation [5]. Preclinical studies have revealed synergistic effects when LSD1 inhibitors are combined with other targeted agents, suggesting that dual or multi-target strategies may overcome single-agent limitations [6].

Beyond epigenetic modulation, apoptosis evasion via anti-apoptotic proteins such as BCL-2 is a hallmark of AML pathogenesis. The BCL-2 inhibitor venetoclax, particularly in combination with hypomethylating agents, has already transformed therapy for older or unfit patients through its potent pro-apoptotic effects [7]. Moreover, IDH1 mutations—present in a significant subset of AML—induce the oncometabolite 2-hydroxyglutarate, impair differentiation, and foster leukemogenesis. FDA-approved IDH1 inhibitors like ivosidenib effectively restore differentiation in IDH1-mutant AML [8].

**Figure 1:**
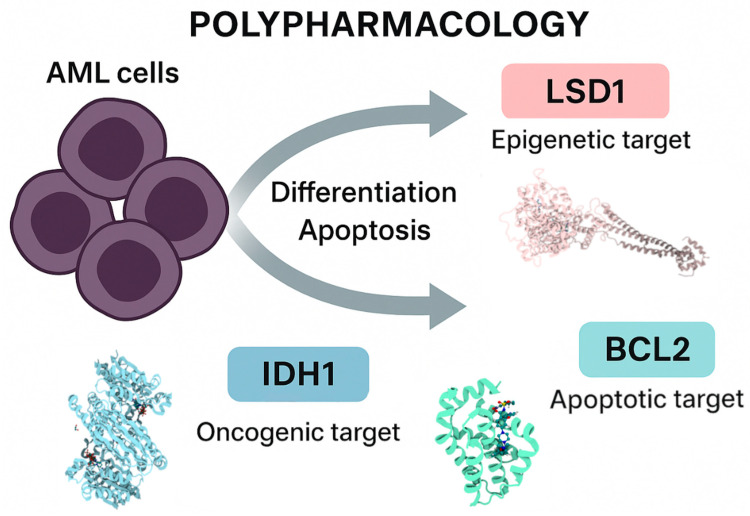
Polypharmacology in Acute Myeloid Leukemia (AML). Schematic representation of AML cells and three critical therapeutic targets: LSD1 (epigenetic target), whose inhibition releases the differentiation block; BCL-2 (apoptotic target), where blockade by venetoclax restores apoptosis; and mutant IDH1 (oncogenic target), whose inhibition reverses the production of the oncometabolite 2-hydroxyglutarate and promotes normal differentiation. Together, these pathways highlight opportunities for polypharmacology-based therapeutic strategies in AML.

Despite these advances, monotherapy approaches often yield limited durability due to compensatory mechanisms and tumor heterogeneity. Thus, there is rising interest in multi-target-directed ligands (MTDLs), single molecules capable of inhibiting several key disease-relevant proteins simultaneously (Figure 1). This approach promises enhanced efficacy, reduced resistance, and simplified pharmacological profiles.

In this study, we report the identification and characterization of three FDA-approved compounds (DB16703, DB08512, and DB16047) with strong binding affinities and favorable interaction profiles across three critical AML targets—LSD1, BCL-2, and mutant IDH1 (R132H). Through comprehensive in silico screening, ADMET profiling, and molecular dynamics simulations, we highlight the potential of these compounds as compelling candidates for multi-target therapeutic intervention in AML, aspiring to meet the evolving needs of differentiation- and apoptosis-based combination therapy.

## 2. Materials and Methods

### 2.1. Protein Structures Preparation

To identify ligands with multi-target potential, we selected three key AML targets: LSD1, BCL2, and mutant IDH1. Their 3D crystal structures were retrieved from the RCSB PDB (Table 1) [9,10,11,12]. PDBFixer was used to prepare the proteins by modeling missing residues, optimizing side chains, adding hydrogens, and removing heteroatoms such as co-crystallized ligands and water molecules. The cleaned structures were then processed with the Meeko package to assign atom types and Gasteiger partial charges, generating PDBQT files suitable for docking with AutoDock Vina v1.2.6 [13].

### 2.2. Ligand Dataset Preparation

DrugBank v5.1.13 was downloaded in SDF format, containing 3D structures of over 10,000 compounds. Initial screening was performed using DataWarrior v6.1.0, which calculated drug-likeness scores; compounds with negative scores were excluded, yielding 3957 drug-like molecules [14]. The remaining compounds were energy-minimized using Open Babel v3.1.0 with the MMFF94 force field (1000 steps) to optimize geometries. Finally, The Forli Lab’s Meeko v0.6.1 was used to assign atom types and Gasteiger partial charges and convert the structures into PDBQT format for docking.

### 2.3. Molecular Docking

Molecular docking was performed using AutoDock Vina to screen the filtered drug-like compounds against the three selected targets [13]. Grid box coordinates were defined in PyMOL (version 3.1) based on the binding sites of the native co-crystallized ligands: LSD1 at 34.38 × 50.25 × 41.00, BCL2 at −11.78 × 10.08 × 8.74, and mutant IDH1 at −28.76 × −101.20 × 23.71 in the x, y, and z directions, respectively. To validate the docking protocol, redocking of the reference ligands was first conducted using the same parameters. The structure-based virtual screening process was then automated using the VinaScreen.py script (https://github.com/yboulaamane/VinaScreen, accessed on 20 July 2025) [15]. To prioritize compounds with multi-target potential, binding scores were ranked separately for each target, and an aggregated rank was computed by summing the individual ranks across all targets.

### 2.4. ADMET Prediction

The pharmacokinetic and toxicity profiles of the top-scoring compounds were predicted using the pkCSM online platform (http://biosig.unimelb.edu.au/pkcsm/, accessed on 25 July 2025 [16]. SMILES representations of each ligand were submitted to evaluate key ADMET properties, including water solubility, Caco-2 permeability, human intestinal absorption (HIA), blood–brain barrier (BBB) permeability, CYP1A2 and CYP2D6 inhibition, total clearance, renal OCT2 substrate status, and AMES toxicity. This in silico profiling allowed early assessment of oral bioavailability, metabolic liability, clearance, and potential toxicity to support hit prioritization.

### 2.5. Molecular Dynamics Simulations

To evaluate the dynamic stability of the top-ranked ligand-protein complexes (LSD1, BCL2, and mutant IDH1), molecular dynamics (MD) simulations were carried out using the GROMACS 2019.3 package [17]. The CHARMM27 force field was employed to describe the proteins [18], and ligand topologies were generated through the SwissParam server [19]. Each complex was solvated in a dodecahedral box with a 1.0 nm buffer using the TIP3P water model, and counterions (Na^+^ and Cl^−^) were added at a physiological concentration of 0.15 mM to neutralize the system. Energy minimization was performed using the steepest descent algorithm until the maximum force reached 1000 kJ/mol/nm [20]. Subsequently, equilibration was achieved in two consecutive steps: a 100 ps NVT simulation at 300 K, followed by a 100 ps NPT simulation at 1 bar using the V-rescale thermostat and Parrinello–Rahman barostat, respectively. Finally, 100 ns production runs were conducted for each complex, with coordinates saved at regular intervals for post-simulation analysis [21]. The resulting trajectories were analyzed to compute backbone RMSD and other structural parameters to assess conformational stability and ligand-induced effects on protein dynamics.

## 3. Results

### 3.1. Docking Protocol Validation

**Figure 2:**
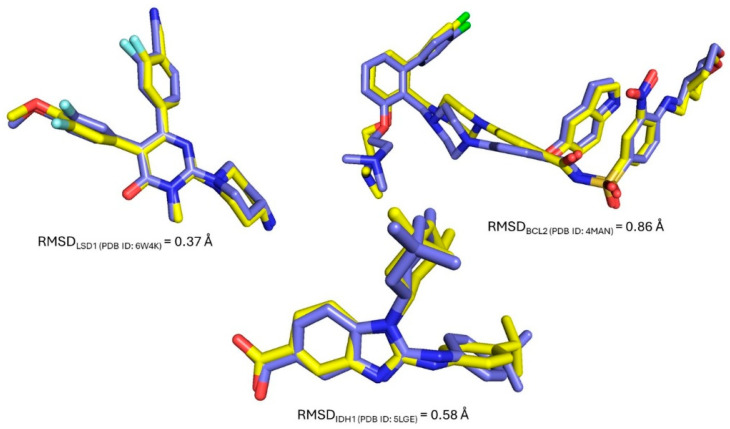
Redocking validation of reference ligands for LSD1, BCL2, and mutant IDH1. Native ligand poses (purple) are overlaid with redocked conformations (yellow).

To validate the docking protocol and assess the reliability of the selected parameters, redocking of the native ligands was performed for each target protein. The co-crystallized ligands were extracted, prepared, and re-docked into their respective binding sites using the same grid coordinates and docking conditions applied in the virtual screening workflow. The resulting docked poses were then aligned with the crystallographic conformations to compute the root-mean-square deviation (RMSD) values, a widely accepted metric for docking accuracy. As shown in Figure 2, the RMSDs were 0.37 Å for LSD1, 0.86 Å for BCL2, and 0.58 Å for mutant IDH1, all falling below the commonly accepted threshold of 2.0 Å for successful redocking [22]. These results indicate that the docking setup can reliably reproduce experimentally observed binding modes, supporting the validity of the structure-based virtual screening results obtained in this study.

### 3.2. Structure-Based Virtual Screening Results

Structure-based virtual screening of 3957 drug-like compounds from DrugBank identified three top-ranking FDA-approved candidates (DB16703, DB08512, and DB16047) with strong binding affinity across the three selected AML targets: LSD1, BCL2, and mutant IDH1 (R132H). Their docking results were compared to known reference ligands for each protein, as summarized in Table 2. For LSD1, all three candidates outperformed the reference ligand (−8.42 kcal/mol), with DB16703 showing the strongest binding (−10.7 kcal/mol), forming multiple hydrogen bonds (THR-335, ALA-539, PHE-560, HIS-564) and hydrophobic contacts (e.g., PHE-538, GLU-559). DB08512 and DB16047 also showed favorable binding energies (−10.28 and −10.54 kcal/mol), engaging key active site residues. In the case of BCL2, while the reference compound had a strong binding score (−10.84 kcal/mol), all three candidates still showed substantial affinity, ranging from −9.39 to −9.84 kcal/mol. Notably, DB08512 formed stable hydrogen bonds with ASN-140, GLY-142, and ARG-143, while DB16047 and DB16703 also interacted with key residues such as ALA-146 and PHE-101. For mutant IDH1 (R132H), the reference ligand exhibited the highest score (−11.09 kcal/mol), but DB08512 closely followed with −10.71 kcal/mol, forming hydrogen bonds with LYS-212A and SER-280B and extensive hydrophobic contacts. DB16703 and DB16047 showed comparable affinities (−10.45 and −10.47 kcal/mol), interacting with residues critical for ligand recognition and catalytic activity.

**Figure 3:**
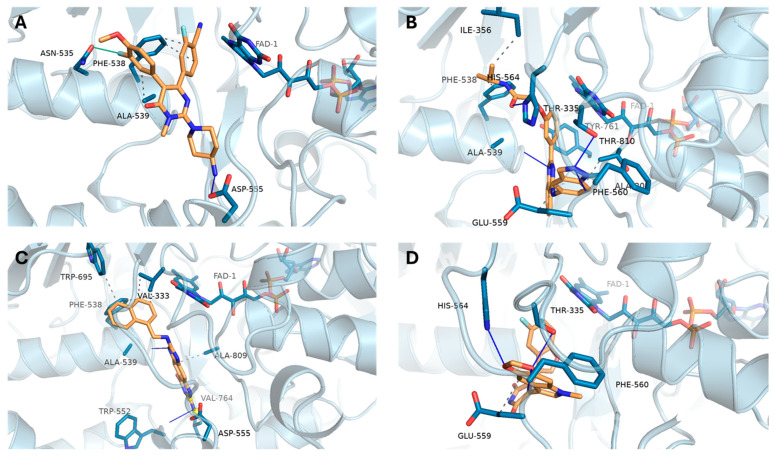
Predicted binding poses and interaction profiles of compounds within the LSD1 active site, visualized using PLIP/PyMOL. (**A**) Reference ligand; (**B**) DB16703; (**C**) DB08512; (**D**) DB16047. Key hydrogen bonds (dashed lines) and hydrophobic interactions with catalytic residues (e.g., PHE-538, ALA-539, HIS-564, and FAD cofactor) are highlighted.

The binding interactions of selected compounds with LSD1 are shown in Figure 3. The reference ligand (Figure 3A) formed key hydrogen bonds with residues such as PHE-538, ALA-539, and ASP-555, anchoring the ligand within the active site near the FAD cofactor. Among the repurposed candidates, DB16703 (Figure 3B) exhibited an enhanced interaction profile, establishing multiple hydrogen bonds with THR-335, HIS-564, and PHE-560, along with hydrophobic contacts involving ILE-356, GLU-559, and others. Similarly, DB08512 (Figure 3C) and DB16047 (Figure 3D) formed stable interactions with catalytic residues and surrounding hydrophobic pockets, supporting their potential as LSD1 inhibitors.

**Figure 4:**
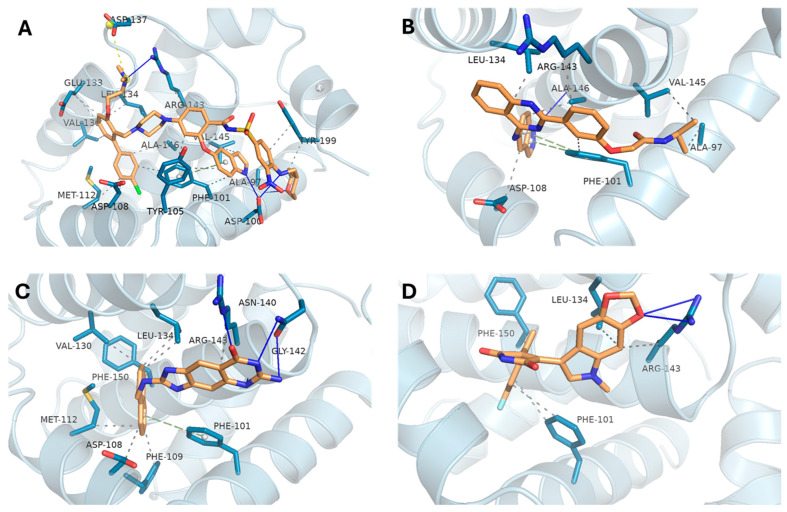
Docking poses and key interactions of compounds with BCL2. (**A**) Reference ligand; (**B**) DB16703; (**C**) DB08512; (**D**) DB16047. Hydrogen bonds and hydrophobic contacts with key residues (e.g., ARG-143, PHE-101, LEU-134) are highlighted.

The docking poses for BCL2 are illustrated in Figure 4. The reference ligand (Figure 4A) engaged deeply within the binding groove, forming several key interactions with ARG-143, TYR-105, PHE-101, and ALA-146. Notably, DB08512 (Figure 4C) closely replicated this interaction network, forming hydrogen bonds with ARG-143, ASN-140, and GLY-142. Both DB16703 (Figure 4B) and DB16047 (Figure 4D) maintained strong hydrophobic interactions with PHE-101 and LEU-134, indicating good binding stability within the BCL2 pocket despite slightly lower affinity scores.

**Figure 5:**
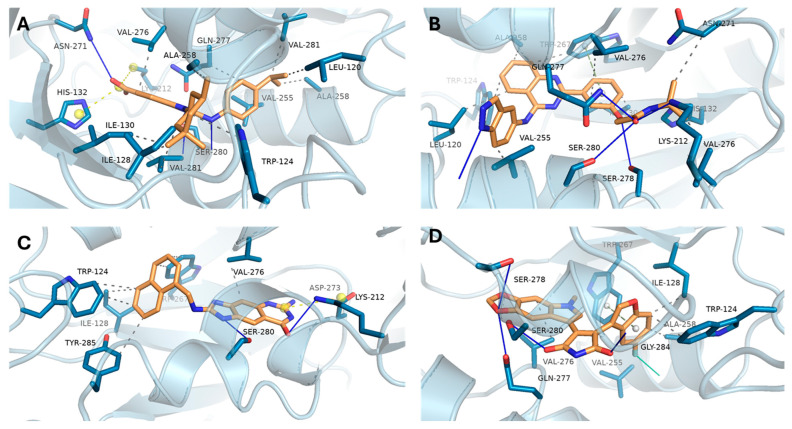
Docking poses and key interactions of compounds with mutant IDH1 (R132H). (**A**) Reference ligand; (**B**) DB16703; (**C**) DB08512; (**D**) DB16047.

For mutant IDH1 (R132H), the interaction profiles are presented in Figure 5. The reference ligand (Figure 5A) demonstrated extensive binding interactions, including hydrogen bonds with SER-280, GLN-277, and ASN-271, and hydrophobic contacts with TRP-124 and ILE-130. DB08512 (Figure 5C) mirrored this pattern, forming key contacts with SER-280, LYS-212, and TYR-285, suggesting a strong and specific fit within the mutant active site. Likewise, DB16703 (Figure 5B) and DB16047 (Figure 5D) engaged with crucial residues such as VAL-276 and GLN-277, supporting their potential as effective IDH1 inhibitors.

### 3.3. ADMET Analysis

All three repurposed compounds exhibited favorable ADMET profiles. They showed good water solubility and high Caco-2 permeability (>0.5), indicating potential for oral absorption. Human intestinal absorption exceeded 91% for all, and BBB permeability values suggest possible CNS penetration, particularly for DB08512 and DB16047 (Table 3). None were predicted to inhibit CYP1A2 or CYP2D6 or act as renal OCT2 substrates. Importantly, all were non-mutagenic (AMES test). Total clearance values were higher than the references, suggesting efficient elimination. Overall, the compounds display promising pharmacokinetic and safety profiles.

### 3.4. Molecular Dynamics Trajectory Analysis

#### 3.4.1. LSD1 Complexes Analysis

**Figure 6:**
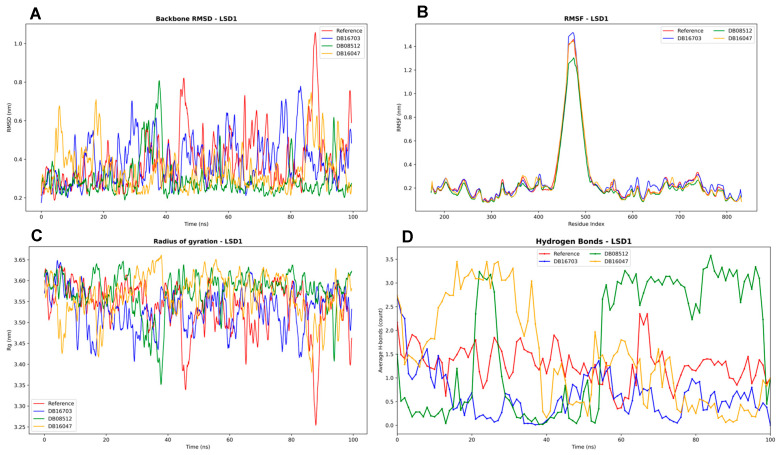
Molecular dynamics simulation analysis of LSD1 in complex with reference and selected ligands (DB16703, DB08512, DB16047) over 100 ns. (**A**) Backbone RMSD showing overall structural stability, with all complexes maintaining fluctuations within ~0.2–1.0 nm. (**B**) RMSF profiles highlighting localized flexibility near residues 480–520, corresponding to the FAD-binding loop region, while the catalytic core remains stable. (**C**) Radius of gyration (Rg) indicating that the overall protein compactness was preserved in all systems. (**D**) Hydrogen bond analysis demonstrating differences in interaction stability across ligands, with DB08512 forming the most persistent H-bonds, while DB16047 and DB16703 displayed greater variability.

The backbone RMSD plots (Figure 6) show that all ligand-bound complexes (DB16703, DB08512, DB16047) and the reference remain largely stable within ~0.2–0.8 nm over the 100 ns simulation. DB16047 (orange) displays some transient deviations toward 0.9–1.0 nm, indicating localized conformational fluctuations, while DB08512 (green) and DB16703 (blue) remain closer to the reference (red). These stable RMSD values suggest that ligand binding does not induce major destabilization of LSD1.

The RMSF analysis reveals low flexibility across most residues, except for a sharp peak near residues 480–520, consistent with the FAD-binding loop and adjacent flexible regions reported in LSD1 crystal structures. The similarity of fluctuation patterns across complexes indicates that ligand binding does not drastically alter residue mobility, though subtle differences may fine-tune stability around the catalytic site.

Rg values remain stable (~3.35–3.65 nm) across all complexes, suggesting that the overall compactness of LSD1 is maintained during simulation. Minor oscillations in DB16703 and DB08512 reflect transient breathing motions, while DB16047 exhibits slightly higher fluctuations. Stability in Rg supports the structural integrity of LSD1 under ligand binding, aligning with the notion that inhibitors stabilize the protein fold without inducing unfolding.

Hydrogen bond profiles differ across ligands. DB08512 maintains ~2–3 stable hydrogen bonds throughout the trajectory, suggesting stronger and more persistent binding interactions. In contrast, DB16703 and DB16047 show more fluctuating H-bond counts, indicating less consistent stabilizing contacts. The reference exhibits fewer stable hydrogen bonds overall, reinforcing the role of ligand binding in enhancing interaction stability.

#### 3.4.2. BCL2 Complexes Analysis

**Figure 7:**
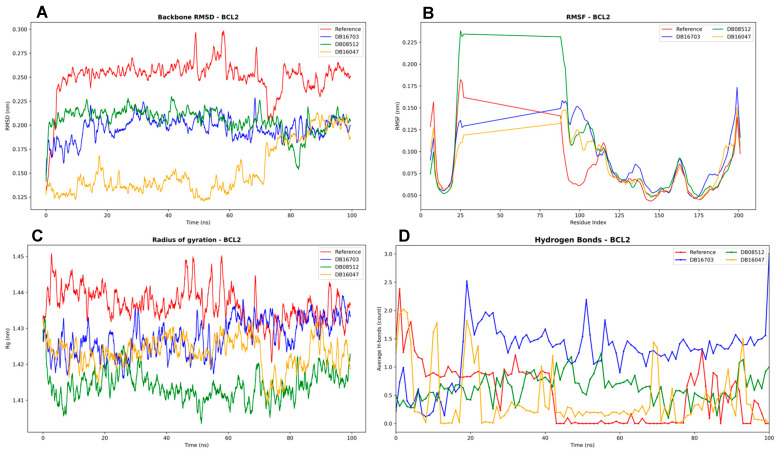
Molecular dynamics simulation analysis of BCL2 in complex with reference and selected ligands (DB16703, DB08512, DB16047) over 100 ns. (**A**) Backbone RMSD profiles showing overall stability, with the reference system displaying the highest fluctuations and DB16047 maintaining the lowest deviations. (**B**) RMSF analysis indicating low residue flexibility (<0.2 nm) across most regions, with localized fluctuations in the N-terminal loop for DB08512. (**C**) Radius of gyration (Rg) demonstrating that all systems remain compact, with DB16047 exhibiting the most stable values. (**D**) Hydrogen bond analysis showing variability among ligands, with DB16703 and DB08512 maintaining ~1–2 persistent H-bonds, while DB16047 relies more on non-polar interactions for stability.

The RMSD profiles (Figure 7) suggest relatively stable complexes, with values fluctuating around 0.22–0.28 nm throughout the 100 ns simulation. The reference shows the highest deviations, whereas DB16047 exhibits the lowest fluctuations, indicating more rigid binding. DB16703 and DB08512 occupy intermediate stability ranges, with minor variations after ~60 ns.

The RMSF analysis highlights differences in local flexibility. Most complexes remain below 0.2 nm, indicating stable residues overall. Notably, DB08512 shows unusually elevated fluctuations in the N-terminal residues (20–70), possibly reflecting unstable binding interactions or loop flexibility in the BH domains, as reported in structural studies of BCL2 family proteins. By contrast, DB16047 and DB16703 align more closely with the reference, indicating minimal destabilization. Importantly, the crystal structure used in this study (PDB ID: 4MAN) represents a truncated form of BCL-2, covering roughly residues 1–166, and lacks the flexible loop domain (FLD) spanning approximately residues 25–90. This FLD region is intrinsically unstructured and unstable, and it is typically absent or disordered in crystallographic structures, including 4MAN, because it does not crystallize well. Consequently, the observed RMSF differences in this segment likely reflect both inherent structural disorder and ligand-induced perturbations rather than stable secondary structure dynamics.

All complexes maintain relatively constant Rg values (~1.41–1.45 nm), confirming that the protein remains compact. The reference shows slightly higher Rg values, suggesting more expansion in the absence of ligand. DB16047 demonstrates consistently lower Rg, implying that this ligand helps preserve structural compactness of BCL2. This supports the RMSD/RMSF results, strengthening the hypothesis that DB16047 stabilizes the overall fold.

Hydrogen bonding analysis reveals significant variability among ligands. DB16703 and DB08512 form a relatively stable network of 1–2 hydrogen bonds over time, whereas DB16047 shows lower counts, suggesting its stabilization may rely more on hydrophobic and van der Waals contacts rather than polar interactions. The reference maintains fluctuating, but generally fewer H-bonds, indicating ligand binding enhances stability by increasing persistent contacts.

#### 3.4.3. IDH1 Complexes Analysis

**Figure 8:**
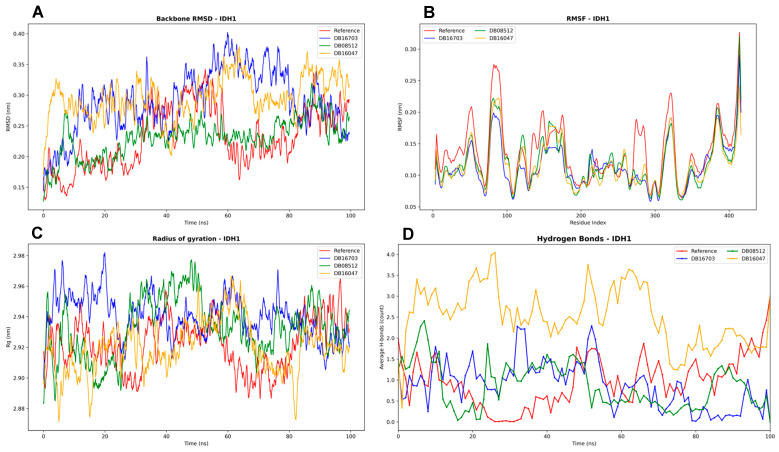
Molecular dynamics simulation analysis of IDH1 in complex with reference and selected ligands (DB16703, DB08512, DB16047) over 100 ns. (**A**) Backbone RMSD profiles showing stable conformations for all complexes, with deviations maintained below 0.40 nm. (**B**) RMSF analysis indicating low residue fluctuations (<0.3 nm) across most regions, with localized flexibility in loop regions between residues 80–120 and 350–400. (**C**) Radius of gyration (Rg) demonstrating that all systems retained compact folds with stable values around 2.92–2.96 nm. (**D**) Hydrogen bond analysis highlighting interaction differences, with DB16047 forming the highest number of persistent hydrogen bonds, while DB08512 showed fewer and more variable interactions.

The stability of IDH1 in complex with the selected ligands (Figure 8) was first evaluated through backbone RMSD analysis. All systems remained stable throughout the 100 ns simulation, with RMSD values below 0.40 nm. The reference complex displayed the lowest deviations, whereas DB08512 and DB16047 exhibited slightly higher fluctuations, suggesting greater conformational adjustments upon ligand binding. These observations indicate that the ligands did not induce significant structural destabilization, with overall conformations remaining within acceptable stability ranges previously reported for IDH1–inhibitor complexes.

To further investigate flexibility at the residue level, RMSF profiles were analyzed. The results showed that fluctuations across the majority of residues remained below 0.30 nm, indicating well-maintained structural rigidity. Localized peaks were observed in loop regions, particularly between residues 80–120 and 350–400, consistent with reported flexible domains of IDH1 that undergo conformational switching relevant to inhibitor binding. Importantly, the RMSF trends were similar across all complexes, suggesting that ligand binding did not significantly perturb the global dynamic behavior of the protein.

Global compactness of the complexes was monitored through the radius of gyration. All systems maintained values at around 2.92–2.96 nm, reflecting a stable and compact fold throughout the trajectory. While DB08512 exhibited slightly higher variability, no complex showed evidence of unfolding.

Finally, hydrogen bond analysis revealed differences in interaction stability across ligands. The reference complex maintained an average of one to two hydrogen bonds, whereas DB16047 consistently formed the highest number of interactions (up to 3–4), suggesting strong and persistent binding. DB08512 displayed the lowest and most fluctuating hydrogen bond count, which may correlate with weaker affinity. DB16703 occupied an intermediate profile, maintaining moderate stability. Consistent hydrogen bond formation, as observed for DB16047, is often associated with improved binding strength and ligand residence time.

Taken together, the MD simulations demonstrate that IDH1 maintains structural stability in all systems. Among the tested compounds, DB16047 appears to achieve the most favorable interaction profile, characterized by stable hydrogen bonding and acceptable conformational stability, whereas DB08512 may represent a weaker binder.

## 4. Discussion

### 4.1. Docking and Molecular Dynamics Insights

Structure-based virtual screening identified three FDA-approved compounds—Belumosudil (DB16703), DB08512, and Elraglusib (DB16047)—as top candidates with predicted binding to LSD1, BCL-2, and mutant IDH1. Docking simulations revealed that each compound could snugly occupy the canonical binding sites of these targets. For example, Belumosudil (originally a ROCK2 inhibitor for chronic GVHD [23]) was predicted to insert into the LSD1 active site (near the FAD cofactor) and form stabilizing interactions analogous to known LSD1 inhibitors. Similarly, all three compounds bound in silico to the BCL-2 BH3-domain groove—spanning key hydrophobic pockets (P1–P4) that normally engage pro-apoptotic BH3 helices—suggesting they may act as BH3 mimetics to displace BCL-2′s client proteins. Notably, docking poses for these drugs in BCL-2′s groove overlapped with Venetoclax’s binding orientation (occupying the P2 and P4 pockets) [24], implying a capacity to trigger apoptosis via BCL-2 antagonism. In the mutant IDH1 (R132H) enzyme, the compounds were predicted to bind at the allosteric pocket that controls the neomorphic activity, potentially blocking the production of the 2-HG oncometabolite. These multi-target binding modes were further validated by MD simulations. Extended 100 ns MD runs demonstrated that each drug-target complex remained conformationally stable, with low RMSD fluctuations and preservation of critical intermolecular contacts. For instance, the Belumosudil–LSD1 complex maintained hydrogen bonding for active-site residues throughout the simulation, indicating a robust and specific interaction. Likewise, in simulations of DB08512 within BCL-2, the compound remained nestled in the hydrophobic groove without dissociation, and Elraglusib bound stably at the IDH1^R132H dimer interface. The convergence of docking and MD results strengthens confidence that these drugs can concurrently engage three disparate AML targets in a biologically meaningful manner. Notably, not one of these compounds has been validated in AML models to date. Belumosudil is approved for chronic graft-versus-host disease but has not been studied in AML. DB08512 is an experimental compound with no clinical use reported, and Elraglusib (9-ING-41) is a GSK-3β inhibitor in trials for other malignancies (e.g., myelofibrosis) without demonstrated efficacy in AML [23,25,26]. Furthermore, Belumosudil’s original indication as a ROCK2 inhibitor and Elraglusib’s role as a GSK-3β inhibitor are acknowledged, underscoring that their repurposing for AML entails known off-target activities [23,25]. These kinase targets could contribute additional effects, for instance, ROCK2 or GSK-3β pathway modulation, which should be carefully evaluated in further preclinical testing.

In parallel, in silico ADMET profiling and known pharmacological data suggest that these compounds possess favorable drug-like characteristics compatible with polypharmacological use. Belumosudil is an orally bioavailable small molecule (~64% oral bioavailability) with a moderate elimination half-life (~19 h) [23], indicating it can achieve and sustain therapeutically relevant plasma levels. Elraglusib (9-ING-41) is likewise a small-molecule GSK-3β inhibitor in clinical trials [25,26], implying it has acceptable pharmacokinetic and safety properties in humans. DB08512, while not previously deployed clinically, showed no violation of key drug-likeness criteria in our predictions, and it lacks structural alerts for overt toxicity (though empirical validation will be needed). The moderate molecular weights (~350–450 Da) and polar surface areas of these compounds fall within ranges conducive to cell permeability, supporting their ability to reach intracellular targets like LSD1 (nuclear) and IDH1 (cytosolic). Together, these ADMET and stability results underscore the viability of repurposing these compounds: not only do they fit the targets of interest, but they also exhibit pharmacological properties suitable for further development as multi-target AML agents.

### 4.2. Polypharmacology in AML: A Multitarget Strategy

The ability of single compounds to modulate multiple oncogenic proteins aligns with the emerging paradigm of polypharmacology in precision oncology. Acute myeloid leukemia is a molecularly heterogeneous disease with dynamic clonal evolution, such that any single-agent therapy can be circumvented by alternate pathways or resistant subclones [27]. Indeed, AML’s heterogeneity and adaptive plasticity demand a broader repertoire of therapeutic molecules or simultaneous blockade of several nodes in leukemia survival networks [27]. Polypharmacology—the deliberate design or use of drugs that act on multiple targets—offers a means to overcome biological redundancy and drug resistance by hitting the cancer from multiple angles at once [28]. This approach can preempt compensatory mechanisms and has the potential to yield more durable responses than one-target “magic bullet” drugs, which tumors often evade [28]. In practical terms, a single multitarget agent could mimic combination therapy while simplifying treatment: reducing drug–drug interactions, cumulative toxicity, and patient non-compliance associated with complex regimens [28]. Our findings exemplify this strategy by identifying compounds that unite epigenetic, apoptotic, and metabolic inhibition in one molecular scaffold. Notably, two of the targets addressed—BCL-2 and mutant IDH1—are individually validated in AML therapy, as evidenced by recent FDA approvals of Venetoclax (BCL-2 inhibitor) and Ivosidenib/Olutasidenib (IDH1 inhibitors) for AML subsets [27]. These successes confirm that both apoptosis induction and metabolic reprogramming can translate to clinical benefit. By integrating those mechanisms with LSD1 inhibition, a multitarget drug could attack AML on three fronts simultaneously. This kind of “combination therapy in a single drug” is especially attractive in AML, where polygenic resistance and clonal heterogeneity have thwarted many single-agent treatments. In summary, the present study’s multi-target approach leverages polypharmacology to potentially surmount two central challenges in AML—therapeutic resistance and disease heterogeneity—thereby embodying a strategic advance in drug repurposing for this aggressive leukemia.

### 4.3. Rationale for Concurrent LSD1, BCL2, and IDH1 Inhibition

Targeting LSD1, BCL-2, and mutant IDH1 together is grounded in a strong biological rationale, as these proteins represent complementary hallmarks of AML pathogenesis. LSD1 (KDM1A) is a lysine demethylase that is often overexpressed in AML and is critical for maintaining the undifferentiated, self-renewing state of leukemic stem/progenitor cells [29]. LSD1 represses myeloid differentiation programs through its epigenetic eraser activity and interactions with transcriptional repressors; accordingly, LSD1 inhibition has been shown to induce differentiation of AML blasts and ablate their clonogenic potential [29]. Early-phase trials of LSD1 inhibitors (e.g., iadademstat/ORY-1001) confirm that pharmacologic LSD1 blockade can drive morphological and molecular differentiation of leukemic cells in patients [29]. However, LSD1 inhibitors as monotherapy have yielded only partial responses, in part because differentiation alone may not eliminate all leukemic cells. This has spurred intense investigation into synergistic partners for LSD1 inhibition [29]. One such partner is BCL-2, a pro-survival protein that AML cells (especially primitive progenitors) rely on to evade apoptosis. BCL-2′s pathological role in AML is underscored by the clinical efficacy of the BH3-mimetic venetoclax, which binds the hydrophobic groove of BCL-2 and liberates pro-apoptotic effectors to trigger cell death [24]. Venetoclax-based regimens have produced high remission rates in older AML patients by ablating the apoptotic blockade, but their durability is limited by resistance mechanisms such as upregulation of alternative anti-apoptotic proteins (MCL-1, BCL-X_L) or metabolic changes in leukemic cells. Strikingly, recent research revealed a direct link between our chosen targets: mutant IDH1-driven AML is unusually dependent on BCL-2. IDH1 mutations (e.g., R132H) produce the oncometabolite 2-hydroxyglutarate (2-HG), which impairs mitochondrial respiration and lowers the threshold for apoptosis; as a result, IDH1-mutant AML cells are selectively sensitive to BCL-2 inhibition [30]. This provides a clear rationale for co-targeting IDH1 and BCL-2—an approach being validated by trials combining IDH1 inhibitors with venetoclax in IDH-mutant AML. Furthermore, IDH1 mutations, present in ~6–10% of AML, enforce an epigenetic state of differentiation arrest (via 2-HG-mediated blockade of TET and other demethylases). Inhibiting mutant IDH1 can relieve this block and promote differentiation of the leukemic clones, as seen with IDH1 inhibitors that often induce terminal myeloid differentiation in responders. Taken together, a therapeutic strategy that simultaneously inhibits LSD1, BCL-2, and mutant IDH1 could yield synergistic effects: LSD1 inhibition unleashes differentiation, IDH1 inhibition restores normal epigenetic and metabolic function, and BCL-2 inhibition ensures that differentiating or metabolically impaired leukemic cells undergo apoptosis. This triple-action mechanism addresses both major facets of AML cell survival—the differentiation blockade and apoptosis resistance—which is expected to produce deeper and more lasting remissions. Indeed, the concept is supported by ongoing combination studies: for example, LSD1 inhibitors are now being tested with venetoclax (and hypomethylating agents) in frontline AML, reflecting the recognition that concurrent epigenetic and apoptotic targeting is highly potent [31]. By achieving this multi-pronged attack with a single repurposed drug, our approach could circumvent the need to administer multiple agents while hitting the same critical nodes in AML biology.

## 5. Limitations and Future Directions

While our computational findings are encouraging, we acknowledge that they are an initial step and come with important limitations. All results were derived from in silico modeling—docking scores and MD simulations are surrogate indicators of binding and do not guarantee biochemical inhibition or cellular activity. Proteins like LSD1 and IDH1 may undergo conformational changes or have co-factor interactions in cells that our models do not fully capture. Additionally, the multitarget nature of these compounds means that their potency against each target may vary; the effective concentration needed to inhibit all three proteins in vivo might be higher than what is clinically achievable or tolerable. Off-target effects, not apparent from our focused simulations, could also emerge given that two of the compounds (Belumosudil and Elraglusib) were initially developed for other protein targets (ROCK2 and GSK-3β, respectively). It is noteworthy that Elraglusib’s known activity against GSK-3β could conceivably add a fourth target in AML cells—GSK-3 inhibition has been shown to synergize with LSD1 inhibition to promote AML differentiation—but this aspect also exemplifies the complexity of polypharmacology, where unintended interactions might be beneficial or detrimental [32].

Therefore, experimental validation is the crucial next step. None of the three candidates has yet shown activity in AML cell studies or clinical settings, especially in the context of venetoclax-resistant or other refractory AML. Therefore, dedicated in vitro and in vivo experiments (e.g., cytotoxicity assays in AML cell lines, including drug-resistant variants, and murine xenograft studies) will be the critical next steps to determine whether the predicted multi-target efficacy translates into actual anti-leukemia activity. We propose comprehensive in vitro studies to confirm that these compounds indeed inhibit LSD1′s demethylase activity, bind and antagonize BCL-2, and block the mutant IDH1 enzyme. Cellular assays in AML models (including IDH1-mutant cell lines and patient-derived blasts) should be conducted to observe the phenotypic effects: do these drugs induce differentiation (e.g., expression of myeloid markers), trigger apoptosis (cleavage of caspases, loss of mitochondrial membrane potential), and reduce 2-HG levels? Crucially, combination index studies could compare a single multitarget drug to the three single-agent combinations to ensure the polypharmacology approach recapitulates or exceeds the efficacy of separate drugs. In vivo, murine xenograft models of AML—particularly an IDH1-mutant AML model—would be informative to test whether, for example, Belumosudil or Elraglusib can impair leukemia progression and extend survival by these mechanisms. Pharmacodynamic assays in such models can confirm target engagement (e.g., changes in H3K4 methylation for LSD1, BAX/BAK activation for BCL-2, and 2-HG reduction for IDH1). Should these repurposed compounds show promising activity, medicinal chemistry optimization might be explored to enhance potency across all targets or minimize any off-target liabilities. Since these agents are either approved or in clinical development, a key advantage is the wealth of existing safety and pharmacokinetic data—this could accelerate translation into clinical trials for AML, bypassing much of the early-phase uncertainty. Nevertheless, careful dose-finding in the new disease context would be required, especially to balance the on-target effects on multiple pathways and avoid unexpected toxicities.

In conclusion, this discussion illustrates that a multitarget drug repurposing strategy could address the complex therapeutic needs of AML. By integrating structure-based design, ADMET profiling, and dynamics simulations, we pinpointed three compounds capable of hitting epigenetic, apoptotic, and metabolic vulnerabilities in concert. This polypharmacological approach aims to overcome the limitations of single-agent therapies by preempting resistance and tackling the disease’s intrinsic heterogeneity. Future preclinical studies will determine whether these computational predictions translate into genuine anti-leukemic efficacy. If they do, the broader significance is profound: it would exemplify how rational repurposing and polypharmacology can yield novel, more effective treatments for AML, potentially improving patient outcomes by delivering combination-like therapy in a single, repositioned drug. Such a strategy, built on the foundations outlined here, could accelerate the development of durable therapies in AML and serve as a paradigm for tackling other refractory cancers through multitarget drug design.

## Figures and Tables

**Table 1:** Summary of selected AML-relevant protein targets, their corresponding PDB structures, reference ligands, and crystallographic resolution.

Protein	Gene	PDB ID	Reference Drug	Resolution
Lysine-specific histone demethylase 1A	*LSD1*	6W4K	Pulrodemstat	2.93 Å
B-cell lymphoma 2	*BCL2*	4MAN	BDBM178570	2.07 Å
Isocitrate Dehydrogenase 1	*IDH1* mutant (R132H)	5LGE	BDBM389289	2.70 Å

**Table 2:** Docking results of reference ligands and selected FDA-approved compounds against LSD1, BCL2, and mutant IDH1 (R132H).

Target	Ligand	Docking Score (kcal/mol)	H Bonds	Hydrophobic
LSD1	Pulrodemstat	−8.42	ASP-555	PHE-538, ALA-539
	DB16703	−10.7	THR-335, ALA-539, PHE-560, HIS-564	ILE-356, PHE-538, GLU-559, TYR-761, ALA-809, THR-810
	DB08512	−10.28	ALA-539, TRP-552, VAL-764	VAL-333, PHE-538, TRP-695, ALA-809
	DB16047	−10.54	THR-335, PHE-560, HIS-564	GLU-559
BCL2	BDBM178570	−10.84	ALA-97, ASP-100, ARG-143	PHE-101, TYR-105, ASP-108, MET-112, VAL-130, GLU-133, LEU-134, ARG-143, VAL-145, ALA-146, TYR-199
	DB16703	−9.63	ALA-146	ALA-97A, PHE-101A, ASP-108A, LEU-134A, ARG-143A, VAL-145A, ALA-146A
	DB08512	−9.84	ASN-140, GLY-142, ARG-143	ASP-108, PHE-109, MET-112, VAL-130, LEU-134, ARG-143, PHE-150
	DB16047	−9.39	ARG-143	PHE-101, LEU-134, ARG-143, PHE-150
IDH1 R132H mutant	BDBM389289	−11.09	ASN-271B, SER-280B, VAL-281B	LEU-120A, TRP-124B, ILE-128B, ILE-130B, VAL-255A, ALA-258A, ALA-258B, VAL-276A, GLN-277A, VAL-281A, VAL-281B
	DB16703	−10.45	HIS-132B, LYS-212A, VAL-255A, GLN-277A, SER-278B, SER-280B	LEU-120B, TRP-124B, ILE-130B, VAL-255A, ALA-258B, TRP-267B, ASN-271B, VAL-276A, VAL-276B
	DB08512	−10.71	LYS-212A, SER-280B	TRP-124B, ILE-128B, TRP-267B, VAL-276A, TYR-285B
	DB16047	−10.47	GLN-277A, SER-278B, SER-280B, GLY-284B	TRP-124B, ILE-128B, ALA-258B, VAL-276A

**Table 3:** In silico ADMET properties of reference ligands and selected FDA-approved compounds predicted using pkCSM.

Ligand	Water Solubility	Caco2 Permeability	HIA	BBB Permeability	CYP1A2 Inhibitior	CYP2D6 Inhibitior	Total Clearance	Renal OCT2 Substrate	AMES Toxicity
Ref-LSD1	−5.07	0.74	91.34	−0.93	No	No	0.20	No	No
Ref-BCL2	−3.84	0.34	96.05	−2.15	No	No	0.09	No	Yes
Ref-IDH1	−6.75	0.81	87.67	0.01	No	No	0.19	No	No
DB16703	−5.56	0.67	92.50	−0.70	No	No	0.95	No	No
DB08512	−4.58	0.53	91.26	−0.50	No	No	1.15	No	No
DB16047	−4.95	1.01	92.31	−0.53	No	No	1.06	No	No

## Data Availability

The original contributions presented in this study are included in the article. Further inquiries can be directed to the corresponding author.

## References

[1] Yashar W.M., Curtiss B.M., Coleman D.J., VanCampen J., Kong G., Macaraeg J., Estabrook J., Demir E., Long N., Bottomly D. (2023). Disruption of the MYC Superenhancer Complex by Dual Targeting of FLT3 and LSD1 in Acute Myeloid Leukemia. Mol. Cancer Res..

[2] Madan V., Koeffler H.P. (2021). Differentiation therapy of myeloid leukemia: Four decades of development. Haematologica.

[3] Yu P.-H., Zhu C.-Y., Kang Y.-Y., Naranmandura H., Yang C. (2025). Mutation in the Unrearranged PML Allele Confers Resistance to Arsenic Trioxide in Acute Promyelocytic Leukemia. Research.

[4] Bercier P., Wang Q.Q., Zang N., Zhang J., Yang C., Maimaitiyiming Y., Abou-Ghali M., Berthier C., Wu C., Niwa-Kawakita M. (2023). Structural Basis of PML-RARA Oncoprotein Targeting by Arsenic Unravels a Cysteine Rheostat Controlling PML Body Assembly and Function. Cancer Discov..

[5] Ravasio R., Ceccacci E., Nicosia L., Hosseini A., Rossi P.L., Barozzi I., Fornasari L., Zuffo R.D., Valente S., Fioravanti R. (2020). Targeting the scaffolding role of LSD1 (KDM1A) poises acute myeloid leukemia cells for retinoic acid–induced differentiation. Sci. Adv..

[6] Pandey M.R., Wang E.S. (2019). Full article: What potential is there for LSD1 inhibitors to reach approval for AML?. Expert Opin. Emerg. Drugs.

[7] Waclawiczek A., Leppä A.-M., Renders S., Stumpf K., Reyneri C., Betz B., Janssen M., Shahswar R., Donato E., Karpova D. (2023). Combinatorial BCL2 Family Expression in Acute Myeloid Leukemia Stem Cells Predicts Clinical Response to Azacitidine/Venetoclax. Cancer Discov..

[8] Watts J.M., Shaw S.J., Jonas B.A. (2024). Looking Beyond the Surface: Olutasidenib and Ivosidenib for Treatment of mIDH1 Acute Myeloid Leukemia. Curr. Treat. Options Oncol..

[9] Kanouni T., Severin C., Cho R.W., Yuen N.Y.Y., Xu J., Shi L., Lai C., Del Rosario J.R., Stansfield R.K., Lawton L.N. (2020). Discovery of CC-90011: A Potent and Selective Reversible Inhibitor of Lysine Specific Demethylase 1 (LSD1). J. Med. Chem..

[10] Souers A.J., Leverson J.D., Boghaert E.R., Ackler S.L., Catron N.D., Chen J., Dayton B.D., Ding H., Enschede S.H., Fairbrother W.J. (2013). ABT-199, a potent and selective BCL-2 inhibitor, achieves antitumor activity while sparing platelets. Nat. Med..

[11] Pusch S., Krausert S., Fischer V., Balss J., Ott M., Schrimpf D., Capper D., Sahm F., Eisel J., Beck A.C. (2017). Pan-mutant IDH1 inhibitor BAY 1436032 for effective treatment of IDH1 mutant astrocytoma in vivo. Acta Neuropathol..

[12] Kouranov A., Xie L., de la Cruz J., Chen L., Westbrook J., Bourne P.E., Berman H.M. (2006). The RCSB PDB information portal for structural genomics. Nucleic Acids Res..

[13] Eberhardt J., Santos-Martins D., Tillack A.F., Forli S. (2021). AutoDock Vina 1.2.0: New Docking Methods, Expanded Force Field, and Python Bindings. J. Chem. Inf. Model..

[14] Sander T., Freyss J., Von Korff M., Rufener C. (2015). DataWarrior: An open-source program for chemistry aware data visualization and analysis. J. Chem. Inf. Model..

[15] Boulaamane Y., Bolivar Avila S., Hurtado J.R., Touati I., Sadoq B.-E., Al-Mutairi A.A., Irfan A., Al-Hussain S.A., Maurady A., Zaki M.E.A. (2025). Computational screening of natural products as tryptophan 2,3-dioxygenase inhibitors: Insights from CNN-based QSAR, molecular docking, ADMET, and molecular dynamics simulations. Comput. Biol. Med..

[16] Pires D.E.V., Blundell T.L., Ascher D.B. (2015). pkCSM: Predicting small-molecule pharmacokinetic and toxicity properties using graph-based signatures. J. Med. Chem..

[17] Park J.-Y., Lee Y., Lee H.J., Kwon Y.-S., Chun W. (2020). In silico screening of GABA aminotransferase inhibitors from the constituents of Valeriana officinalis by molecular docking and molecular dynamics simulation study. J. Mol. Model..

[18] Bjelkmar P., Larsson P., Cuendet M.A., Hess B., Lindahl E. (2010). Implementation of the CHARMM Force Field in GROMACS: Analysis of Protein Stability Effects from Correction Maps, Virtual Interaction Sites, and Water Models. J. Chem. Theory Comput..

[19] Zoete V., Cuendet M.A., Grosdidier A., Michielin O. (2011). SwissParam: A fast force field generation tool for small organic molecules. J. Comput. Chem..

[20] Al-Khafaji K., Taskin Tok T. (2020). Molecular dynamics simulation, free energy landscape and binding free energy computations in exploration the anti-invasive activity of amygdalin against metastasis. Comput. Methods Programs Biomed..

[21] Boulaamane Y., Jangid K., Britel M.R., Maurady A. (2023). Probing the molecular mechanisms of α-synuclein inhibitors unveils promising natural candidates through machine-learning QSAR, pharmacophore modeling, and molecular dynamics simulations. Mol. Divers..

[22] Warren G.L., Andrews C.W., Capelli A.M., Clarke B., LaLonde J., Lambert M.H., Lindvall M., Nevins N., Semus S.F., Senger S. (2006). A Critical Assessment of Docking Programs and Scoring Functions. J. Med. Chem..

[23] Cutler C., Lee S.J., Arai S., Rotta M., Zoghi B., Lazaryan A., Ramakrishnan A., DeFilipp Z., Salhotra A., Chai-Ho W. (2021). Belumosudil for chronic graft-versus-host disease after 2 or more prior lines of therapy: The ROCKstar Study. Blood.

[24] Birkinshaw R.W., Gong J., Luo C.S., Lio D., White C.A., Anderson M.A., Blombery P., Lessene G., Majewski I.J., Thijssen R. (2019). Structures of BCL-2 in complex with venetoclax reveal the molecular basis of resistance mutations. Nat. Commun..

[25] Shaw G., Cavalcante L., Giles F.J., Taylor A. (2022). Elraglusib (9-ING-41), a selective small-molecule inhibitor of glycogen synthase kinase-3 beta, reduces expression of immune checkpoint molecules PD-1, TIGIT and LAG-3 and enhances CD8+ T cell cytolytic killing of melanoma cells. J. Hematol. Oncol..

[26] Mahalingam D., Saeed A., Powell S.F., Huerta M., Sahai V., Coveler A.L., Davis E.J., Steeghs N., Mulcahy M., Raufi A.G. (2025). Phase II study of elraglusib (9-ING-41), a GSK-3β inhibitor, in combination with gemcitabine plus nab-paclitaxel in previously untreated metastatic pancreatic cancer. ESMO Open.

[27] Andresen V., Gjertsen B.T. (2017). Drug Repurposing for the Treatment of Acute Myeloid Leukemia. Front. Med..

[28] Abdelsayed M. (2025). AI-Driven Polypharmacology in Small-Molecule Drug Discovery. Int. J. Mol. Sci..

[29] Deb G., Wingelhofer B., Amaral F.M.R., Maiques-Diaz A., Chadwick J.A., Spencer G.J., Williams E.L., Leong H.-S., Maes T., Somervaille T.C.P. (2020). Pre-clinical activity of combined LSD1 and mTORC1 inhibition in MLL-translocated acute myeloid leukaemia. Leukemia.

[30] Chan S.M., Thomas D., Corces-Zimmerman M.R., Xavy S., Rastogi S., Hong W.-J., Zhao F., Medeiros B.C., Tyvoll D.A., Majeti R. (2015). Isocitrate dehydrogenase 1 and 2 mutations induce BCL-2 dependence in acute myeloid leukemia. Nat. Med..

[31] National Cancer Institute (NCI) (2025). Phase 1 Trial of Iadademstat in Combination with Venetoclax and Azacitidine in Patients with Treatment Naive AML.

[32] Hosseini A., Dhall A., Ikonen N., Sikora N., Nguyen S., Shen Y., Amaral M.L.J., Jiao A., Wallner F., Sergeev P. (2025). Perturbing LSD1 and WNT rewires transcription to synergistically induce AML differentiation. Nature.

